# The Effect of Parathyroidectomy on Risk of Hip Fracture in Secondary Hyperparathyroidism

**DOI:** 10.1007/s00268-017-4000-0

**Published:** 2017-03-24

**Authors:** Elin Isaksson, Kerstin Ivarsson, Shahriar Akaberi, Andreas Muth, Gunnar Sterner, Prütz Karl-Göran, Naomi Clyne, Martin Almquist

**Affiliations:** 1 Department of Clinical Sciences, Lund University, Skåne University Hospital, Malmö, Sweden; 2 Department of Urology, Lund University, Skåne University Hospital, Malmö, Sweden; 3 Department of Child and Adolescent Psychiatry, Lund University, Lund, Sweden; 4 Department of Nephrology, Lund University, Skåne University Hospital, Lund, Sweden; 5 Endocrine and Sarcoma Surgery, Department of Surgery, Institute of Clinical Sciences at the Sahlgrenska Academy, University of Gothenburg, Gothenburg, Sweden; 6 Department of Clinical Sciences, Faculty of Medicine, Lund University, Skåne University Hospital, Lund, Sweden; 7 Department of Nephrology, Lund University, Skåne University Hospital, Malmö, Sweden; 8 Department of Internal Medicine, Helsingborg Hospital, Helsingborg, Sweden; 9 Department of Surgery Section of Endocrine and Sarcoma, Lund University, Skåne University Hospital, Lund, Sweden

## Abstract

**Background:**

Secondary hyperparathyroidism increases the risk for fractures. Despite improvement in medical therapy, surgical parathyroidectomy (PTX) often becomes necessary, but its effect on risk of fractures is not clear. Our aim was to study the effect of parathyroidectomy on the risk of hip fractures in patients on dialysis or with a functioning renal graft at time of parathyroidectomy.

**Design:**

In a cohort of 20,056 patients on dialysis or with functioning renal allograft, we identified 590 patients who underwent parathyroidectomy between 1991 and 2009. Of these, 579 were matched with 1970 non-PTX patients on age, sex, cause of renal disease and functioning renal allograft or not at the time of PTX or at the corresponding time for non-PTX patients (*t*). We calculated the risk for hip fracture after PTX using crude and adjusted Cox proportional hazards regressions, adjusting for time in renal replacement therapy before *t*, time with functioning renal allograft before and after *t*, comorbidity at *t* and a hip fracture before *t*.

**Results:**

The adjusted hazard ratio (95% confidence interval) for hip fracture was 0.40 (0.18–0.88) for PTX patients, compared to non-PTX patients. When analyses were performed separately for sex, only women had a lower risk of hip fracture after PTX compared to non-PTX patients. The risk of hip fracture after PTX was similar in patients with or without functioning renal allograft at time for PTX.

**Conclusion:**

Parathyroidectomy is associated with a lower risk of hip fracture in female patients with secondary hyperparathyroidism.

## Introduction

Secondary hyperparathyroidism (sHPT) is common among patients on chronic renal replacement therapy (RRT) [1]. SHPT increases the risk for fractures [2–5], and patients on RRT have a higher risk of fractures than the general population [6]. Patients on dialysis or with a renal transplant have different, additional risk factors for fractures than the general population such as immobility during time spent on dialysis, treatment with corticosteroids, abnormal levels of PTH [7]. In sHPT, the mineral metabolism is disturbed and levels of parathyroid hormone (PTH) are increased. This is treated medically, but in patients with severe sHPT who fail to respond to medical therapy, surgical removal of parathyroid glands is required. Surgical parathyroidectomy (PTX) reduces plasma levels of PTH calcium and phosphate [8, 9], but the effect of PTX on fracture risk has not been well documented [9]. There are indications that PTX improves bone density and bone pain [10, 11]. A previous study found a reduction in risk of fractures [12], but included only patients on dialysis. Patients with a renal transplantation are an important group within the RRT population, and the effects of PTX on fractures in these patients are not known. Therefore, we aimed to study the effect of PTX on the risk of hip fractures in patients on dialysis and in patients with a functioning renal graft at time of PTX in a nationwide, population-based cohort.

## Methods

### Study cohort

We performed a matched index-referent study within a cohort consisting of all patients in the Swedish Renal Registry (SRR) between 1 January 1991 and 31 December 2009. Registration in the SRR is mandatory for all patients who start dialysis or receive a renal transplant in Sweden. All dialysis- and renal transplantation units in Sweden are affiliated with SRR, and its coverage is almost 100% [13]. In this study, RRT was defined as treatment with maintenance dialysis or a having a functioning renal transplant.

### Identification of PTX, comorbidity and fractures

To retrieve date of PTX and hospital discharge diagnoses, we linked data from the Swedish Inpatient Registry to SRR. The Swedish Inpatient Registry has a national coverage of nearly 100% since 1987 and has high validity [14, 15]. We defined PTX as total or subtotal parathyroidectomy. The dates of PTX were then compared with data from the Scandinavian Quality Register for Thyroid Parathyroid and Adrenal Surgery (SQRTPA). Since its start in 2004, this registry collects information on all thyroid and parathyroid surgeries in approximately 90% of all units performing this surgery in Sweden [16]. Discharge diagnoses were translated from ICD7-9 to ICD10 by using conversion tables from the Swedish National Board of Health and Welfare [17]. Diagnoses were used to create comorbidity groups according to Charlson [18], using the algorithm described by Quan et al [19]. ICD10 codes used to identify hip fractures were: “S72.0, S72.1, S72.2, S72.3, S72.4, S72.7, S72.8 and S72.9”. To validate the diagnostic codes, we also collected codes for surgical intervention for hip fracture using ICD10 codes: “NFJ 09, NFJ19, NFJ29, NFJ39, NFJ39, NFJ49, NFJ59, NFJ69, NFJ79, NFJ89 and NFJ99”. We chose hip fracture as our main event since it usually leads to hospitalization and thus can be studied through the inpatient register.

### Exclusions

During the study period, there were 20,056 patients in the SRR. Of these, we excluded 175 patients due to: PTX before registration in SRR (*n* = 130), censoring (death) at date of initiation of SRR (*n* = 27), errors in reporting of patient information (*n* = 15) and death at date of parathyroid surgery (*n* = 3).

### Matching

After entry in SRR, 590 patients underwent PTX. These patients were matched with one to five patients who had not undergone PTX. Patients were matched using the following criteria: birth year (in 10 year categories), sex, cause of renal failure in categories (autosomal dominant polycystic kidney disease, diabetes mellitus, glomerulonephritis, nephrosclerosis, pyelonephritis and other/unknown) and if the patient had a functioning renal allograft at the calendar date of PTX (or at the corresponding time for non-PTX patients, *t)*. Fracture-free survival time was calculated by assigning both the index and referent patients the calendar date of PTX of the index patient, hereafter referred to as *t.* The matched reference patient was required to be alive on this date and to have the same transplantation status as the cases at the calendar date of PTX. Eleven PTX patients could not be matched. The completed matched set consisted of 2549 patients. The same cohort and matched set were recently used by our group to study survival after PTX [20].

### Statistical analyses

For descriptive data, numbers and column per cent are reported for categorical variables and means and standard deviations (SD) for continuous variables. We calculated incidence rates of hip fractures in the whole cohort, as well as for the matched set of PTX and non-PTX patients using time from start in RRT until first hip fracture, death or end of follow-up 31 December 2009, whichever occurred first. We calculated incidence rates for sex, age at start of RRT (in 4 categories: <55 years, 55–65 years, 65–75 years and >75 years) and parathyroidectomy or renal transplantation at any time during the time of observation separately. Time to hip fracture after PTX was calculated with the Kaplan–Meier method, yielding survival curves. Crude and adjusted Cox proportional hazard models were created to compare hazard ratios with 95% confidence intervals (CI) for hip fractures between PTX and non-PTX patients in the matched set. In the adjusted model, we used Charlson index score at *t* as a continuous co-variate, accumulated time in RRT before *t* as a categorical co-variate in three categories (0–2 years, 2–4 years, >4 years) using 0–2 years as reference, and hip fracture before *t* as a categorical co-variate using no hip fracture as reference. Time with a functioning graft before *t* was used as a categorical co-variate in three categories (none, 1–3 years, >3 years), using none as reference. Time with a functioning graft after *t* was used as a categorical co-variate in three categories (none, 1–4 years, >4 years) using none as reference. The time intervals differ between these variables due to unequal distribution of patients. Cox regressions were performed for the PTX and matched non-PTX patients, separately in men and women, separately in patients with and without a history of hip fracture and separately in patients with and without a functioning renal allograft at the calendar date of PTX (*t*). We considered results being statistically significant if *P* < 0.05. All statistical analyses were made using STATA version 12 (StataCorp LP, College station, USA).

## Results

### Demographics and patient characteristics

Patient characteristics of the whole cohort and the matched set of PTX and non-PTX patients are shown in Table 1. Patients in the whole cohort were older at the start of RRT, more often male, and had more often diabetes mellitus as cause of renal failure and were less likely to have received a renal transplant, compared with patients in the matched set. PTX and non-PTX patients in the matched set were similar to each other, both regarding matched and unmatched variables, Table 1. In the matched set of PTX and non-PTX patients, we found a total of 87 hip fractures after *t* and a total of 70 surgical procedures related to hip fracture after *t*. There were 79 patients who had suffered from hip fracture before *t*, of these there were 43 female and 36 male patients. In the PTX group, 22 patients (4%) had a prior hip fracture compared with 57 (3%) in the non-PTX group. Median follow-up was 4 years and 7 months.

**Table 1:** Patient characteristics

Factor	Whole cohort (*n* = 20,011)	Matched PTX and non-PTX patients (*n* = 2549)	
		PTX (*n* = 579)	Non-PTX (*n* = 1970)
Age (years) at start in RRT	*62.8 (16.5)*	*50.1 (14.3)*	*50.4 (14.3)*
Age at PTX (or corresponding date for non-PTX patients = *t*)	–	*54.1 (13.6)*	*54.0 (13.7)*
Sex			
Female	7131 (35.6)	297 (51.3)	975 (49.5)
Male	12,880 (64.4)	282 (48.7)	995 (50.5)
Time on RRT at PTX (*t*) (years)	–	*4.0* (*3.2*)	*3.5* (*3.4*)
Cause of ESRD			
ADPKD	1572 (7.9)	84 (14.5)	293 (14.9)
Diabetes mellitus	4843 (24.2)	89 (15.4)	317 (16.1)
Glomerulonephritis	3182 (15.9)	179 (30.9)	642 (32.6)
Nephrosclerosis	3651 (18.2)	57 (9.8)	175 (8.9)
Pyelonephritis	1009 (5.0)	48 (8.3)	118 (6.0)
Other and unknown	5754 (28.8)	122 (21.1)	425 (21.5)
Number of transplantations			
0	14,951 (74.7)	214 (37.0)	752 (38.2)
1	4686 (23.5)	298 (51.4)	1096 (55.6)
2	347 (1.7)	58 (10.0)	114 (5.8)
3	23 (0.1)	8 (1.4)	7 (0.3)
4	4 (<0.1)	1 (0.2)	1 (0.1)
Functioning allograft at PTX (*t*)	–	156 (27.0)	736 (37.3)
Charlson comorbidity score at PTX (*t*)	–	*1.2 (1.6)*	*1.4 (1.8)*
Hip fracture before PTX (*t*)	–	22 (4.0)	57 (3.0)

*Mean (standard deviation)*, numbers (percent), *PTX* parathyroidectomy, *RRT* renal replacement therapy, *ESRD* end-stage renal disease, *ADPKD* autosomal dominant polycystic kidney disease

### Fracture incidence

The unadjusted incidence rate for hip fractures in the total cohort was 11.0/1000 person years [95% confidence interval (CI) 10.3–11.8] with a total of 79,542 person years observed. The overall unadjusted incidence rate for hip fracture is 2.0/1000 person years in Sweden [16]. The incidence rates in the matched set of PTX/non-PTX patients were overall lower compared to the total cohort. Fracture incidences in the total cohort and in the matched set of PTX and non-PTX patients are summarized in Table 2.

**Table 2:** Incidence rate/1000 person years (95% CI) of hip fractures in Swedish patients on renal replacement therapy

Factor	Whole cohort		Matched PTX and non-PTX patients			
			PTX		No PTX	
	*n* (%)	Incidence rate	*n* (%)	Incidence rate	*n* (%)	Incidence rate
All	20,011 (100)	11.0 (10.3–11.8)	579 (100)	4.1 (2.7–6.2)	1970 (100)	6.1 (5.0–7.4)
Male	12,880 (64)	9.3 (8.5–10.2)	282 (49)	4.5 (2.5–7.9)	995 (51)	4.4 (3.2–6.1)
Female	7131 (36)	14.1 (12.8–15.6)	297 (51)	3.7 (2.0–7.0)	975 (49)	7.8 (6.1–10.0)
Age at start of RRT						
<55	5481 (27)	3.8 (3.2–4.5)	350 (60)	3.0 (1.7–5.4)	1181 (60)	3.6 (2.6–4.8)
55–65	3788 (19)	10.0 (8.6–11.7)	139 (24)	2.6 (0.8–8.0)	499 (25)	7.9 (5.5–11.5)
65–75	5432 (27)	19.1 (17.0–21.5)	75 (13)	15.5 (7.4–32.6)	216 (11)	18.5 (12.0–28.3)
>75	5310 (27)	30.1 (26.8–33.8)	15 (3)	14.8 (2.1–105.3)	74 (4)	35.7 (19.2–66.3)
PTX	676 (4)	4.7 (3.2–6.7)	–	–	–	–
No PTX	19,335 (96)	11.6 (10.8–12.4)	–	–	–	–
No renal transplantation at any time during observation	14,951 (75)	20.7 (19.2–22.2)	214 (37)	11.6 (7.0–19.2)	752 (38)	14.1 (10.8–18.5)
Renal transplantation during observation	5060 (25)	3.5 (3.0–4.1)	365 (63)	1.7 (0.8–3.6)	1218 (62)	3.7 (2.7–4.9)

*PTX* parathyroidectomy, *RRT* renal replacement therapy, – not applicable

### Effect of PTX on risk of fracture

**Fig. 1:**
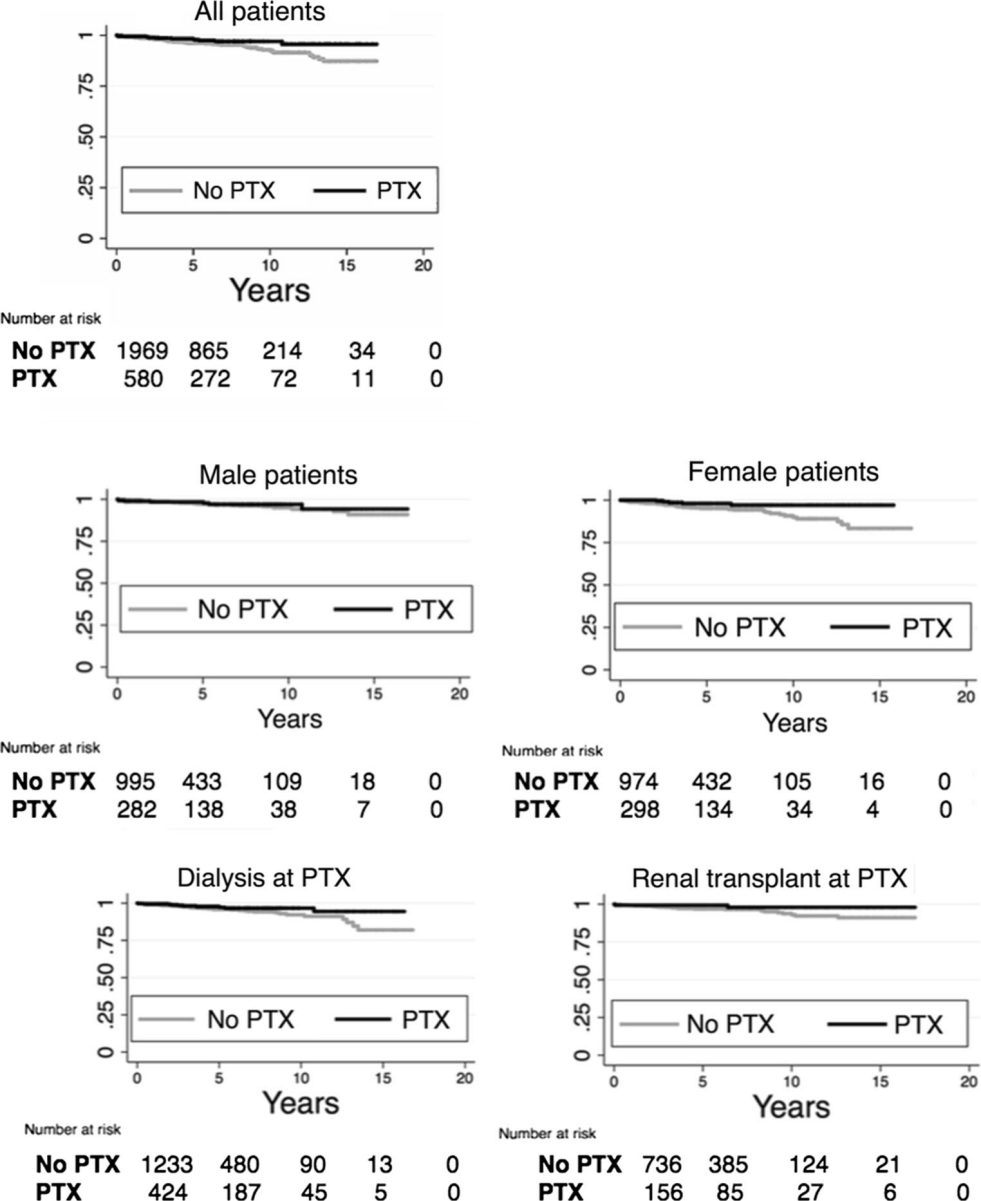
Kaplan–Meier hip fracture-free survival estimate for PTX and non-PTX patients. Time in years to first event. Separate graphs for all patients, female and male gender, dialysis at time of PTX, functioning renal transplant at time of PTX, no hip fracture before PTX and hip fracture before PTX. *PTX* parathyroidectomy

**Fig. 2:**
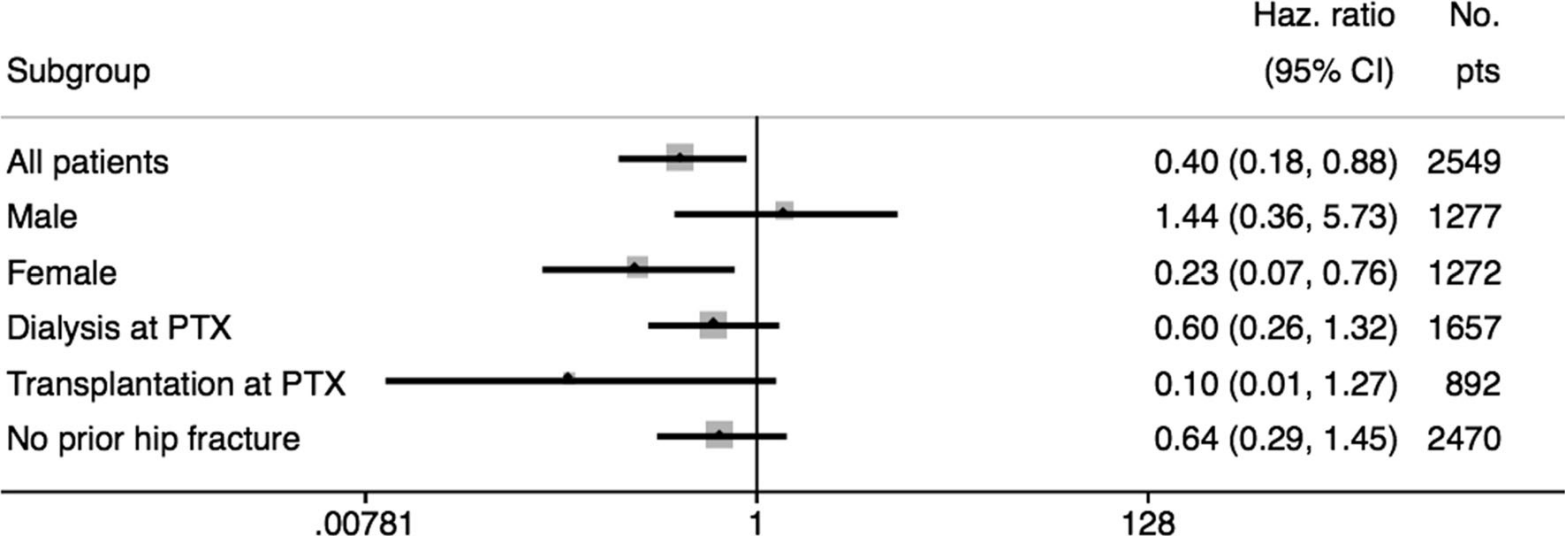
Forest plot of Cox proportional hazards (95% CI) over risk of hip fracture after PTX in subgroup analyses cases compared to references. Adjusted for time in RRT before PTX/t, time with a renal transplant before PTX/t, Charlson comorbidity score at PTX/t, time with a renal transplant after PTX/t and hip fracture before PTX/t. *PTX* parathyroidectomy

The results of the Kaplan Meier curves are shown in Fig. 1. Analyses were made separately for PTX and non-PTX patients, female and male patients, patients with a functioning graft and patients on dialysis at time of PTX and for patients with and without a prior hip fracture. The results of the Cox proportional hazards model are shown in Table 3. After adjusting for hip fracture before *t* comorbidity at *t*, accumulated time on RRT before *t*, time with a functioning graft before *t* and time with a functioning graft after *t* we found a significantly lower hazard ratio (HR) of 0.40 (95% CI 0.18–0.88) for hip fractures in PTX patients compared with non-PTX patients. We also found an association between time with a functioning graft after *t* and risk of hip fracture. Patients with more than 3 years with a functioning graft after *t* had a lower hazard ratio for hip fracture both in the crude and adjusted analyses. Hip fracture before *t* had the strongest association with hip fracture after *t* both in crude and adjusted analyses with an adjusted HR of 5.05 (95% CI 1.57–16.23). Subgroup analyses with the same Cox proportional hazards model are summarized in Fig. 2. When performing analyses separately in men and women, we found a strong association with PTX and lower risk of hip fracture in women (compared to women who had not undergone PTX) with an adjusted HR of 0.23 (95% CI 0.07–0.76). However, in male patients, there was no such association Fig. 2.

**Table 3:** Risk for hip fracture after parathyroidectomy (PTX) patients compared to references, Cox proportional hazards regression, HR (95% CI)

Factor		All (*n* = 2549)	
		Crude	Adjusted
PTX	No	Ref	Ref
	Yes	0.61 (0.31–1.18)	0.40 (0.18–0.88)
Charlson index score		0.97 (0.80–1.18)	0.94 (0.76–1.16)
Hip fracture before *t*	No	Ref	Ref
	Yes	4.86 (1.74–13.59)	5.05 (1.57–16.23)
Cum. time in RRT before PTX	0–2 years	Ref	Ref
	2–4 years	1.51 (0.69–3.30)	1.32 (0.57–3.06)
	>4 years	2.33 (1.05–5.14)	2.40 (0.92–6.21)
Cum. time with graft before PTX	None	Ref	Ref
	1–3 years	3.31 (0.55–20.12)	5.28 (0.68–40.75)
	>3 years	3.05 (0.44–21.13)	2.73 (0.28–26.79)
Cum. time with graft after PTX	None	Ref	Ref
	1–4 years	0.81 (0.25–2.56)	0.88 (0.26–3.04)
	>4 years	0.19 (0.06–0.66)	0.19 (0.05–0.70)

*RRT* renal replacement therapy, *t* = date for PTX (or corresponding date for non-PTX patients)

## Discussion

Patients who underwent PTX had a lower risk of hip fracture compared with non-PTX patients matched on age, sex, cause of renal disease and whether the patient had a functioning renal graft at the date of PTX (or corresponding date, *t*, for non-PTX patients). After adjusting for hip fracture before *t*, time on RRT before *t*, comorbidities at *t*, time with a functioning graft before *t* and time with a functioning graft after *t* the hazard ratio (95% CI) for hip fracture was 0.40 (0.18–0.88). As expected, we found a strong correlation between prior hip fracture and new hip fracture as well as between hip fracture and time spent with a functioning graft after *t*. Other variables in the model were not associated with hip fracture risk. In subgroup analyses for sex, we found that the lower risk of hip fracture in PTX patients only was observed in female patients. The reduced risk of hip fracture in PTX patients was also observed by Rudser et al. [12] who studied a population of patients on dialysis with no prior fracture and found a hazard ratio of 0.68 (95% CI 0.54–0.86) for fracture in patients treated with PTX compared with patients with no PTX. They found no sex differences in the risk of fracture after PTX. Ishani et al. studied 4435 patients with chronic renal failure who underwent PTX and compared one-year fracture rate before PTX with 1 year after PTX. They found a non-significant decrease in the fracture rate in PTX patients. However, they did not report whether there were sex differences in fracture risk after PTX [21]. There are several potential explanations for the lower risk of hip fracture after PTX in women compared with men in our study. Female patients have a higher fracture risk in general as well as in end-stage renal disease [6]. Women on dialysis have higher levels of PTH than men [22] and are more likely to undergo PTX [23, 24]. A more aggressive histological pattern has been shown in female compared with male patients undergoing PTX for sHPT [25]. Furthermore, Cheng et al. [26] found a worse bone mineral metabolism prior PTX in women than in men. Thus, women seem to suffer from more severe sHPT than men, which left untreated may carry a greater risk of hip fracture. Hence, women might gain more from PTX in relation to fracture risk. When excluding patients with a prior hip fracture, the adjusted hazard ratio for PTX patients was non-significant compared to non-PTX patients. This implies that patients with manifest osteoporosis have a greater effect of PTX. Bone disturbances in CKD patients are complex, and many factors contribute to increased fracture risk [27]. There are therefore many possible explanations as to why PTX might reduce the risk of hip fracture. Elevated PTH is associated with increased osteoclastic and osteoblastic activity and high bone turnover [28] with defects in the bone as a result. PTX reduces the level of PTH in the majority of cases [8]. Mazzaferro et al. [29] examined markers of bone formation and degradation before and after PTX in patients with renal failure and found decreased markers of bone degradation and increased markers for bone formation after PTX. By reducing PTH, the high bone turnover state will likely be less pronounced, which can contribute to better bone quality. Both calcium and phosphate plasma levels drop after PTX and patients can suffer from “hungry bone syndrome” caused by rapid re-uptake of minerals by the bone [30]. These changes might have a positive effect for skeletal health which is favourable for fracture outcome. We believe the inclusion of transplanted patients in the present study strengthens the validity of the results. Transplanted patients have different risk factors for fractures, such as treatment with corticosteroids, but we tried to take this into account by adjusting for time spent with a functioning transplant both before and after time for PTX (or corresponding time in controls). The unadjusted incidence rate of a new hip fracture during the observation period in the total cohort was 11.0/1000 person years (9.3 for men and 14.1 for women). Prior findings by Alem et al. [6] in an end-stage renal disease cohort in the USA found an incidence rate of 7.45 in men and 13.63 in women which is in line with our findings. The incidence rate was lower in the matched set of PTX and non-PTX patients which implies that our selected study group has better bone health and might have a better overall health compared to the overall end-stage renal disease population. There are limitations to our study. We did not have information on levels of PTH, calcium, phosphate and renal function. We also lacked data on traditional risk factors for fractures such as medications, body mass index, hormonal status and smoking. Selection bias cannot be excluded, despite the matching procedure and adjustment for potential confounding factors. Strengths in this study include the truly population-based design. The national registries contain information on virtually all RRT patients in Sweden. This lessens the burden of bias due to regional differences in clinical practice. The long follow-up time includes different eras of sHPT treatment and PTX frequency. Data from the Inpatient Registry used to define PTX, co-variates and fracture endpoints have been shown to be reliable [14, 15]. We believe that the fact that we examined hip fractures (that usually require hospitalization and in most cases surgery) makes the results robust. Thus, we conclude that parathyroidectomy was associated with a reduced risk of hip fracture in female patients with sHPT.
